# Turning over a green leaf: integrating light signals toward chloroplast establishment

**DOI:** 10.1093/plphys/kiag482

**Published:** 2026-08-14

**Authors:** Anniek Oosterwijk, Gabriela Toledo-Ortiz, Andrés Romanowski, Charlotte M M Gommers

**Affiliations:** Laboratory of Plant Physiology, Wageningen University & Research, Droevendaalsesteeg 1, 6708PB Wageningen, The Netherlands; Laboratory of Molecular Biology, Wageningen University & Research, Droevendaalsesteeg 1, 6708PB Wageningen, The Netherlands; Laboratory of Molecular Biology, Wageningen University & Research, Droevendaalsesteeg 1, 6708PB Wageningen, The Netherlands; Laboratory of Plant Physiology, Wageningen University & Research, Droevendaalsesteeg 1, 6708PB Wageningen, The Netherlands

## Abstract

Light is indispensable for transitioning from a heterotrophic to a photoautotrophic state. Etiolated seedlings, developed in darkness, are primed for rapid chloroplast development upon light exposure. In this transition, dark-adapted etioplasts differentiate into functional chloroplasts through a tightly regulated process controlled at multiple levels. Light steers gene transcription and translation, as well as posttranslational modifications of both plastid- and nuclear-encoded chloroplast and photosynthetic proteins. Light is sensed not only by dedicated nuclear photoreceptors like phytochromes and cryptochromes, but also directly by plastids themselves. For example, incoming light drives chlorophyll metabolism, which initiates a cascade of structural and metabolic changes. The shaping of functional chloroplasts involves adjustments to the membrane lipid composition and the incorporation of photosynthetic proteins, contributing to the internal membrane structure for the buildup of the photosystems. In this update, we review our current understanding of integrated light signals in plastids and the nucleus that drive the multi-level transition of non-photosynthetic etioplasts toward fully functioning chloroplasts.

## The etioplast-to-chloroplast transition occurs during seedling establishment

**Figure 1:**
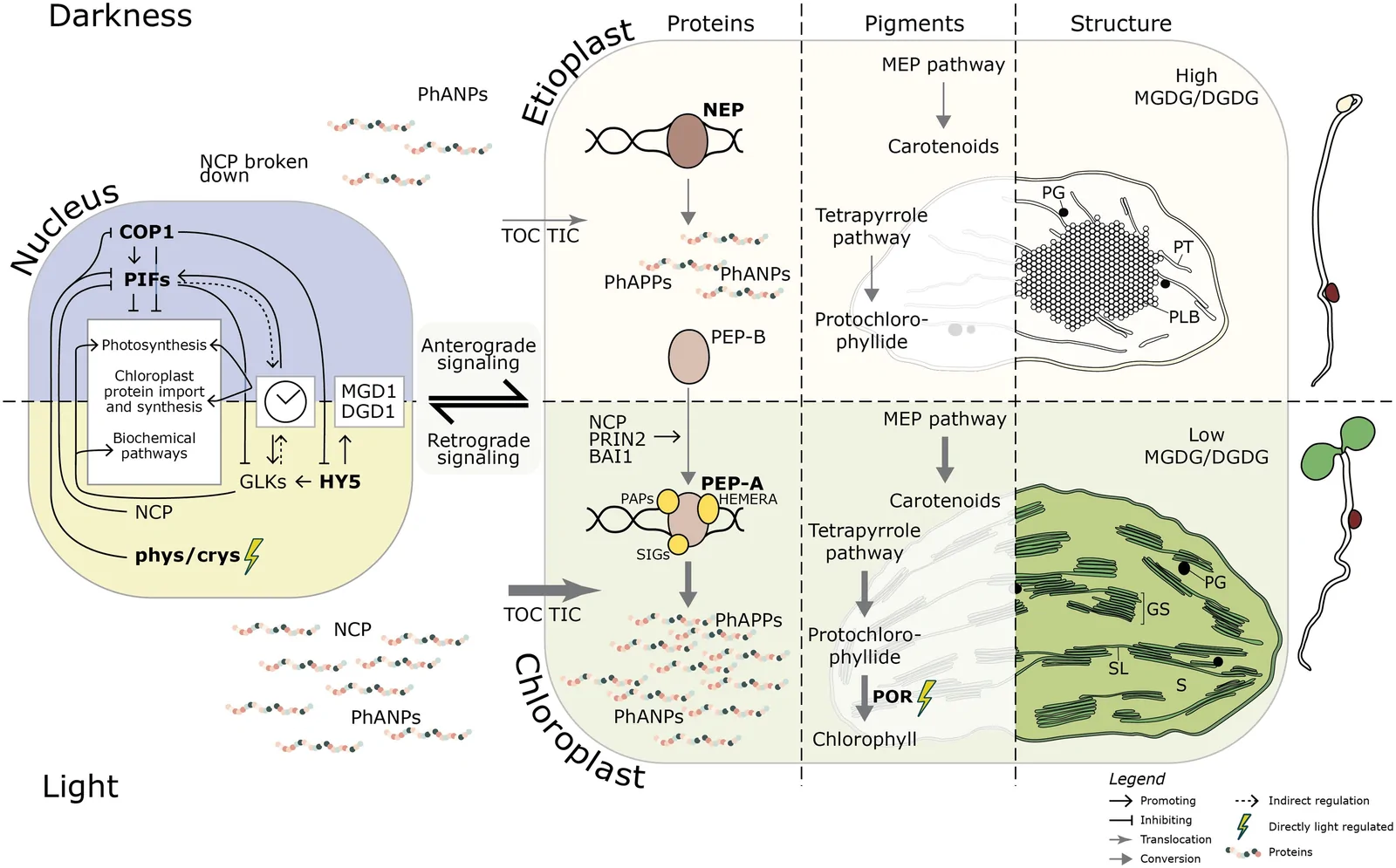
Schematic representation of light-induced regulatory processes during etioplast-to-chloroplast development. During de-etiolation, a broad range of processes in both the nucleus and the plastid undergo reprogramming in response to light, resulting in the development from etioplast to chloroplast, and from etiolated to green and photomorphogenic seedling. In the nucleus, light-activated phytochromes and cryptochromes inactivate CONSTITUTIVE PHOTOMORPHOGENESIS1 (COP1) and PIFs, directly and/or via NCP. Inhibition of COP1 and PIFs releases transcriptional activation of genes encoding for proteins involved in biochemical pathways (such as enzymes involved in the tetrapyrrole pathway and PSY involved in carotenoid synthesis), transcriptional regulation via interaction with the PEP, such as SIGs and PAPs, and PhANPs. Light-activated nuclear transcription factors involved in the upregulation of these genes are GOLDEN2-LIKEs (GLKs) and HY5. GLKs and PIFs are directly regulated by the circadian clock (clock symbol). PIFs, in turn, provide feedback to the clock. The clock is also regulated by chloroplast-to-nucleus retrograde signaling. PhANPs are produced in the cytosol and imported into the plastid via the TOC/TIC complexes. In the plastid, the major transcription machinery switches from a nuclear-encoded polymerase (NEP) to PEP upon the transition to light, and the total Photosynthesis-Associated Plastid-Encoded Protein (PhAPP) content increases. Pigments, needed for photosynthesis, are produced upon light activation of POR, which catalyzes the conversion from protochlorophyllide to chlorophyll (see Fig. 2). The light-driven protein, pigment, and lipid composition allows for formation of the typical grana stack structure, characterizing a photosynthetically active chloroplast. For clarity, we have omitted several intermediate steps and cofactors. Abbreviations: BAI1 = “Albino” in Chinese; DGD1, DGDG synthase; GS, grana stack; MGD1, MGDG synthase; PG, plastoglobulus; PRIN2, PLASTID REDOX INSENSITIVE2; PT, prothylakoids; S, stroma; SL, stroma lamella.

Plants use incoming light as a signal to guide their development toward optimal photon absorption and, consequently, optimal photosynthesis. After a brief heterotrophic phase during seedling establishment, the transition to light marks the start of a photoautotrophic lifestyle, in which photosynthesis delivers energy for growth, development, and homeostasis. In case angiosperm seeds germinate under a soil layer in the dark, seedlings prepare for the upcoming light acclimation in a process called skotomorphogenesis. Skotomorphogenic seedlings are characterized by elongated hypocotyls, an apical hook, and pale cotyledons (Gommers and Monte 2018). Their cells contain non-photosynthetic etioplasts: these dark-adapted plastids develop from proplastids in the embryo and are fully prepared to rapidly establish the photosynthetic machinery as soon as light is perceived. Etioplasts are characterized by the crystalline prolamellar body (PLB), high levels of protochlorophyllide (the precursor for chlorophyll), and the inability to perform photosynthesis (Fig. 1) (Adam et al. 2011). After light exposure, seedlings undergo photomorphogenic development: hypocotyls stop elongating, hooks and cotyledons unfold and, maybe most importantly, etioplasts develop into photosynthetically active chloroplasts. Chloroplasts typically contain stacked thylakoid membranes, high chlorophyll levels, and active photosynthesis (Fig. 1). The transition from etioplasts to chloroplasts is a 2-step process: in the first 24 h after light exposure, the internal chloroplast structure for the photosynthetic machinery is established, followed by chloroplast proliferation (Pipitone et al. 2021). The structural development phase is tightly regulated by signals from within the plastid, as well as from the nucleus. In this review, we summarize our current understanding of this light-driven etioplast-to-chloroplast development and function during the seedling transition from darkness to light, and how this requires tightly controlled multi-organellar signaling pathways on molecular, structural, and functional levels.

## Light controls the plastid proteome

A fully functional chloroplast contains between 2,100 and 3,600 unique proteins, many of which are also present in etioplasts (Leister 2003; Armarego-Marriott et al. 2019). In *Arabidopsis thaliana*, only a small fraction—88 proteins—are encoded by the plastid genome (Li and Chiu 2010). A complex machinery of plastid and nuclear (Box I) transcriptional regulation, translation, posttranscriptional and posttranslational modifications (PTMs), plastid protein import, and circadian timing (Box II) is required to maintain the optimal combination of plastid and nuclear proteins inside the plastid and to ensure establishment of functional chloroplasts from etioplasts (Fig. 1).

Box I **Dark and light signaling in the nucleus**

In darkness, the nucleus tightly controls the functional status of the etioplast. The dark-stable transcription factors PIFs repress photomorphogenesis at various levels. PIF1 and PIF3 prevent overaccumulation of the chlorophyll precursor protochlorophyllide in the dark by inhibiting the expression of enzymes in the tetrapyrrole biosynthesis pathway, such as GENOMES UNCOUPLED4, GUN5, GluTR1, and PORC (Stephenson et al. 2009; Toledo-Ortiz et al. 2014). PIFs repress another class of plastid pigments, the carotenoids, via repression of the major biosynthesis enzyme PSY, which results in high carotenoid levels in the Arabidopsis *pif-quadruple* (*pifq*) mutant (Toledo-Ortiz et al. 2010). At the same time, PIF3 binds to and represses the expression of several genes encoding components of the light-harvesting complexes (Liu et al. 2017). Consequently, dark-grown *pif1* and *pif3* mutants overaccumulate protochlorophyllide, overexpress *PhANG*s, and have partly developed chloroplasts that contain prothylakoid membranes (Huq et al. 2004; Stephenson et al. 2009; Liu et al. 2017). Another repressor of chloroplast development is CONSTITUTIVE PHOTOMORPHOGENESIS1 (COP1): a central regulator of seedling development in darkness. This E3 ligase complex protein stabilizes PIFs, but targets positive regulators of photomorphogenesis, such as HY5, for degradation (Osterlund et al. 2000; Bauer et al. 2004; Kathare et al. 2020). Arabidopsis *cop1* mutants express a photomorphogenic phenotype in darkness, accompanied by chloroplast-like plastids, which lack a prolamellar body (Deng and Quail 1992).

Upon light exposure, nuclear photoreceptors activate signaling pathways that promote de-etiolation, including the coordination of chloroplast development, photopigment accumulation, and buildup and tuning of the photosynthetic machinery for efficient photosynthesis. In particular, the red and far-red light-sensitive phytochromes and the blue light–sensitive cryptochromes play important roles in the progression of chloroplast development and regulation of photosynthetic gene expression (Griffin and Toledo-Ortiz 2022). Photoreceptors coordinate growth with plastid function and deliver environmental information, including light quality, diurnal, and circadian cues, to optimize plastid performance (Jan et al. 2022).

Phytochromes and cryptochromes inactivate PIFs and the COP1 complex, releasing photomorphogenesis-promoting factors. Transcription factors GOLDEN2-LIKE 1 and 2 (GLK1 and GLK2, respectively) are 2 PIF-repressed regulators of chloroplast development (Oh and Montgomery 2014; Martín et al. 2016). GLK1 and GLK2 promote the expression of *PhANG*s, chlorophyll, and carotenoid biosynthesis genes. Disassembly of the COP1 complex causes the stabilization of transcription factors HY5 and HY5 HOMOLOGUE, which promote *PhANG* expression directly, and via transcriptional induction of *GLK1* and *GLK2* (Zhang et al. 2024).

Box II **Circadian timing in the etioplast-to-chloroplast transition**

The circadian clock is an internal timekeeping mechanism that allows plants to anticipate daily environmental changes and coordinates biological processes from gene expression to development (Inoue et al. 2018; Harmer 2025). Although there is no direct mechanistic evidence that the clock regulates the etioplast-to-chloroplast transition, a growing body of work supports circadian control of processes required for greening.

The clock is already functional in etiolated seedlings: rhythmic expression of clock genes in dark-grown Arabidopsis is detectable within 48 h after seed imbibition (Salome et al. 2008). Classic greening assays in barley further showed that de-etiolation proceeded more rapidly when initiated at the circadian phase of maximal (aggregate) chlorophyll a/b-binding genes’ mRNA levels (Beator and Kloppstech 1993), indicating time of day is a variable in this process. Nuclear chloroplast development regulators such as the PIFs and GLKs (Box I) are tightly clock-controlled through transcriptional and chromatin protein interactions (Soy et al. 2016; Martín et al. 2018; Li et al. 2020; Zhang et al. 2020; Tachibana et al. 2024; Tian et al. 2024).

At the plastid level, circadian control is extensive: up to 70% of chloroplast-encoded genes show circadian regulation, mediated in part by rhythmic SIGMA (SIG) factors that convey nuclear timing information to plastid transcription. The best characterized is dawn-phased *SIG5*, which, in a blue light/cry1-dependent manner, directs transcription of *Photosystem II D2* (Noordally et al. 2013; Belbin et al. 2017). It has been postulated that other rhythmic SIG factors, such as dusk-phased *SIG3* and *SIG4* or dawn-phased *SIG1*, may communicate specific time-of-day information to different chloroplast genes (Noordally et al. 2013; Dodd et al. 2015).

*PhANG*s are also circadian-regulated: ∼80% show rhythmic expression in at least 1 dataset (Romanowski et al. 2020; Hwang et al. 2022). Key examples include *PORA*; *HEMA1*; *CHLH/GUN5*, a Mg–chelatase subunit; and *CAB2*, a major light-harvesting protein (Millar et al. 1995; Matsumoto et al. 2004). This gating is likely adaptive to reduce ROS production upon light availability.

Chloroplasts also feed status information back to the circadian clock through retrograde pathways involving carbohydrates, photosynthetic electron flow, and tetrapyrrole intermediates (Box III) (Dodd et al. 2015). The evidence highlights the circadian clock's importance in modulating various processes essential for the etioplast-to-chloroplast transition. However, direct mechanistic links from a developmental timing perspective remain to be addressed.

Illumination affects the transcription of nearly one-third of nuclear genes, including many encoding chloroplast-targeted proteins (Ma et al. 2001). Photosynthesis protein complexes contain plastid- and nuclear-encoded proteins and require coordinated expression and continuous bidirectional communication between the 2 organelles. Anterograde pathways transmit information from the nucleus to the plastids, whereas retrograde pathways (Box III) convey information on plastid status back to the nucleus. Within this regulatory framework, nucleus-localized photoreceptors, particularly phytochromes and cryptochromes, play a central role in aligning photosynthesis with environmental cues. Acting through light-responsive transcription factors (Box I), these photoreceptors orchestrate the etioplast-to-chloroplast transition through expression of *Photosynthesis-Associated Nuclear Genes* (*PhANGs*), including those encoding light-harvesting complexes (LHCs), Calvin–Benson cycle enzymes, and pigment biosynthetic enzymes (Berry et al. 2013). These *PhANGs* are additionally regulated by the circadian clock (Box II).

Box III **Plastid to nucleus retrograde communication and metabolic feedback**

The integration of photoreceptor signaling with chloroplast-derived retrograde communication forms a central axis of plant development and environmental adaptation. Biogenic retrograde signals refer to those emitted by plastids during early steps of chloroplast biogenesis.

Beyond ROS (eg, singlet oxygen ^1^O_2_), other plastidic metabolites serve as signals to modulate nuclear gene expression, ensuring that the synthesis of photosynthetic and plastidic proteins aligns with plastid needs (Pfannschmidt 2010). Photoreceptor pathways that promote chloroplast development (Box I) are counterbalanced by retrograde ROS signals that tune chloroplast development, repress photosynthetic gene expression, and activate protective responses (Pogson et al. 2008).

Identified plastid-derived signals include tetrapyrroles, heme, plastid-produced bilins, methylerythritol cyclodiphosphate (MEcPP) and other apocarotenoids, 3′-phosphoadenosine-5′-phosphate (PAP), β-cyclocitral (β-CC), carbohydrates, and redox cues from the ccccplastoquinone pool, a molecule involved in the electron transport chain during photosynthetic light reactions (Chi et al. 2015a; Lee and Kim 2024; León et al. 2025). Photoreceptor signaling and retrograde signaling are deeply interconnected: photoreceptors adjust nuclear transcriptional networks in anticipation of changing light conditions, while retrograde signals provide feedback on the plastid's physiological status to fine-tune or override photoreceptor-driven outputs (Jan et al. 2022). MEcPP stabilizes phytochrome B and suppresses PIF activity, reprogramming nuclear transcription (Jiang et al. 2019), while PAP, produced during oxidative stress as a byproduct of sulfur metabolism, inhibits nuclear exo-ribonucleases (XRN2,3) mediated RNA decay. This inhibition leads to the stabilization of transcripts and activation of ∼25% of the high-light responsive transcriptome, including photoprotective genes such as those involved in non-photochemical quenching and antioxidants (Estavillo et al. 2011; Gigolashvili et al. 2012). Oxidized β-carotene derivatives, β-CC, and β-cyclocitric acid enhance photooxidative tolerance (D’Alessandro et al. 2018), and ROS and oxylipin-derived signals under high light reprogram nuclear transcription to boost photoprotection (Griffin and Toledo-Ortiz 2022).

Mutants affecting nuclear transcription in response to inhibitors of pigment biosynthesis or plastid transcription, known as gun mutants, have revealed key integration points (Pfannschmidt 2010). In particular, GUN1 integrates photoreceptor inputs via PIFs and GLKs to fine-tune photomorphogenesis and photosynthetic gene expression (Martín et al. 2016; Tokumaru et al. 2016). Metabolic feedback also connects chloroplast and photoreceptor signaling. Sugars and organic acids regulate *PhANG* expression via the HY5/PIF module, coordinating pigment synthesis with photosystem assembly and stress responses (Toledo-Ortiz et al. 2014). Elevated sugar levels suppress phytochrome-HY5 signaling to restrict growth, whereas HY5 can coordinate carbon and nitrogen metabolism with potential impacts on photosynthetic capacity (Chen et al. 2016). Collectively, PIFs integrate light cues with plastid-derived signals to balance chloroplast biogenesis with energy demands (Hernández-Verdeja et al. 2020), while HY5 mediates retrograde signals to activate stress-responsive and antioxidant genes, ensuring that nuclear-encoded photosynthetic proteins remain synchronized with functional plastids (Hernandez-Verdeja and Strand 2018).

Nucleus-encoded chloroplast proteins are synthesized in the cytosol and mainly imported via the nuclear-encoded Translocon at the Outer and Inner envelope membrane of Chloroplasts (TOC and TIC, respectively) complexes, fueled by an ATPase import motor (Ling and Jarvis 2015; Liang et al. 2024). Degradation of specific TOC/TIC complex components by the proteasome allows for selective protein import and steers the plastid proteome composition during the establishment of functional chloroplasts (Ling et al. 2012; Ling et al. 2019; Wan et al. 2023). For example, the nuclear-encoded TOC/TIC complex protein TOC33 is ubiquitinated by the SUPPRESSOR OF PPI1 LOCUS1 (SP1), SP2, and CELL DIVISION CYCLE48 (CDC48) ubiquitin ligase complex and subsequently degraded (Ling et al. 2019, 2012). Recent work shows that MAPKK kinase CONSTITUTIVE TRIPLE RESPONSE 1 (CTR1), known for its role in ethylene sensing, phosphorylates TOC33 in an ethylene-independent way, thereby preventing its degradation, which is essential for chloroplast development (Chien and Yoon 2025). More kinases likely fulfill similar roles, and more TOC proteins are regulated by these complexes. More work is needed to understand the exact regulation of plastid protein import by cytosolic degradation.

Protein import is also regulated at the inside of the plastid envelope. Loss of components of the inner membrane transporter, such as TIC100, in Arabidopsis leads to reduced protein import and seedling lethality (Kikuchi et al. 2013; Loudya et al. 2022). Impaired protein import is sensed and communicated to the nucleus via a key player in plastid-to-nucleus retrograde signaling, GENOMES UNCOUPLED 1 (GUN1) (Box III), and causes the inhibition of *PhANG* expression (Loudya et al. 2022). GUN1 also contributes to chloroplast establishment through additional mechanisms related to protein import. It interacts with cpHSC70-1, an ATP-dependent protein import chaperone, potentially enhancing the chloroplast's protein import capacity (Wu et al. 2018; Wu et al. 2019). Interestingly, GUN1 is only stable in etioplasts and during early chloroplast establishment in the light, making it a specific light-steered regulator of the plastid proteome composition (Hernández-Verdeja et al. 2022). It remains unknown if, and how, light directs the import of proteins into the chloroplast via other translocon complexes.

In addition to protein import, TIC subunits might have other roles. As shown in tobacco, overexpression of the chloroplast gene encoding TIC214 led to only a mild increase in protein levels, indicating strong posttranslational regulation. Nevertheless, this mild upregulation strongly inhibited chloroplast-encoded photosynthetic protein accumulation, while nuclear-encoded protein accumulation was hardly affected. This suggests that TIC214 plays an additional role in the regulation of chloroplast transcription (Wang et al. 2025).

Beyond protein import, chloroplast function also depends on proteins encoded within the plastid genome itself. The plastid genome (the plastome) encodes indispensable building blocks for photosynthesis, as well as transcription and translation of the plastome (Berry et al. 2013; Dobrogojski et al. 2020). At a very early stage in development, the import of nuclear-encoded RNA polymerase into the plastid is essential for gene transcription in the etioplasts (Hricová et al. 2006). At that moment, the plastid-encoded RNA polymerase (PEP) complex most likely consists of only the core proteins, the so-called PEP-B, although fully assembled PEP complexes have been found in etiolated Arabidopsis seedlings as well (Ji et al. 2021). PEP-B is active in etioplasts and consists of only the core proteins. Upon light transition, over 50 proteins associate with PEP to form PEP-A (Liere et al. 2011; Yagi and Shiina 2014). As the chloroplast matures under the influence of light, PEP-A becomes more prevalent and generates around 88% of the plastid transcripts (Hanaoka et al. 2005; Zhelyazkova et al. 2012).

In Arabidopsis, PEP assembly relies on anterograde signals that are controlled via the light-driven, phytochrome-mediated degradation of phytochrome-interacting factors (PIF) 1 and 3 (PIF1 and PIF3, respectively) in the nucleus (Box I). The dual-targeted nuclear and plastidic protein REGULATOR OF CHLOROPLAST BIOGENESIS (RCB) acts as a cofactor of phytochrome B in PIF degradation and thereby indirectly drives PEP assembly in the plastid (Yoo et al. 2019). A similar nuclear role is known for HEMERA, which in addition integrates into the plastid-localized PEP complex to control *Photosynthesis-Associated Plastid Gene* (*PhAPG*) expression (Nevarez et al. 2017). NUCLEAR CONTROL OF PEP ACTIVITY (NCP), another dual-targeted nuclear and plastidic noncatalytic thioredoxin-like protein, similarly operates in phytochrome signaling by promoting nuclear PIF1 and PIF3 degradation and by facilitating PEP complex assembly in plastids (Yang et al. 2019). Interestingly, cellular targeting of NCP is directly steered by light as well. In darkness, PIF1 and PIF3 trigger transcription from an alternative transcriptional start site, resulting in a short version of NCP, which is degraded in the cytosol. Upon illumination, PIFs are degraded and a long version of NCP, containing a chloroplast localization peptide, is transcribed. NCP is further processed in the chloroplast, where it promotes PEP-A assembly during de-etiolation (Lee et al. 2025). BAI1, a nucleus–plastid C2H2 zinc-finger transcription factor, further exemplifies the coordination of nuclear and plastid transcription during chloroplast development in the light (Qin et al. 2025). As a component of the plastid transcriptionally active chromosome, BAI1 interacts with HEMERA to support PEP assembly and directly binds nuclear *RbcS* and plastid *RbcL* promoters to coordinate Rubisco biogenesis; its loss abolishes chloroplast formation and is lethal. In addition, WHIRLY proteins that localize in the nucleus and the plastids bind single-stranded DNA and RNA and support plastid genome stability, retrograde signaling (Box III), and photosynthetic performance, as evidenced by reduced growth and chlorophyll accumulation in *whirly* mutants (Taylor et al. 2023).

Nuclear-encoded sigma (SIG) factors are key proteins in PEP-A for transcription initiation that provide promoter specificity for plastid-encoded transcripts (Chi et al. 2015b). Two out of 6 PEP-associated SIGs, namely SIG2 and SIG6, are crucial in chloroplast development (Kanamaru et al. 2001; Ishizaki et al. 2005). Molecular models reveal that SIGs associate with the large PEP-A complex without steric hindrance from other components (do Prado et al. 2024; Vergara-Cruces et al. 2024). The expression of *SIG* genes is under PIF control in the nucleus and thus tightly linked to phytochrome and cryptochrome signaling cascades (Box I) (Hwang et al. 2022).

Another class of nuclear-encoded PEP subunits is the PEP-associated proteins (PAPs) whose expression is rapidly and strongly induced by light (Liebers et al. 2018). At least 14 PAPs play a crucial role in the light transition. Single PAP knockout plants exhibit an albino phenotype caused by impaired chloroplast structure, even though they all have different functions (Steiner et al. 2011; Ahrens et al. 2025). For example, PAP1 and PAP7 are essential for DNA binding, and PAP2 binds specific RNA sequences (Wang et al. 2024; Wu et al. 2024). PAP11 is suggested to be involved in the binding of SIGs to the PEP (Vergara-Cruces et al. 2024). In general, PAPs are important for the structural integrity of the PEP complex and for maintaining its transcriptionally active state (Vergara-Cruces et al. 2024). Besides their role in transcriptional regulation, PAPs protect transcripts against reactive oxygen species (ROS; PAP4 and PAP9) (Favier et al. 2021). Although it remains under debate whether PLASTID REDOX INSENSITIVE2 (PRIN2) is classified as a PAP, it is essential for PEP activation, interacts with PAP10, and loss of PRIN2 results in pale seedlings (Díaz et al. 2018; Ahrens et al. 2025). Specifically, during de-etiolation and the onset of photosynthesis, PRIN2 is reduced via the ferredoxin-thioredoxin reductase system, which triggers PRIN2 monomerization and subsequent activation, and results in activation of the PEP complex (Díaz et al. 2018). This redox-dependent activation of PEP-A is important to secure *PhAPG* upregulation during light-induced chloroplast development.

In addition, plastid-encoded RNA undergoes a combination of prokaryotic and eukaryotic posttranscriptional processing. Plastid transcripts are produced as polycistronic sequences, similar to bacteria (Bock 2007). After the symbiotic event, however, eukaryotic processes such as RNA editing, intercistronic cleavage and trimming of 5′ and 3′ ends, and cis*-* and trans*-*splicing steps were acquired and are under the control of nuclear-encoded but plastid-acting RNA-binding proteins (Stern et al. 2010; Barkan 2011; Germain et al. 2013).

Pentatricopeptide repeat proteins (PPRs), with over 450 members, are 1 of the largest protein families in Arabidopsis and play a major role in RNA metabolism in plastids (Barkan and Small 2014). They cleave intercistronic RNA regions and are involved in the 5′ end stabilization (Stern et al. 2010; Barkan 2011). At least 20 PPR proteins are involved in plastid-encoded transcript splicing (Wang et al. 2021), but many more remain uncharacterized to date (Zhang et al. 2017). Recently, a couple of PPRs were characterized in more detail. These are essential for chloroplast development, and knockout causes seedling lethality (Zhang et al. 2017; Yuan et al. 2019; Wang et al. 2021, 2020). However, how their expression and function are regulated remains unclear (Wang et al. 2021).

RNases E and J are the main endoribonucleases in chloroplasts and act as intercistronic cleaving factors, cleaving polycistronic sequences into monocistronic ones (Hotto et al. 2020). RNases E and J are essential for chloroplast development and thereby the transition to autotrophic growth (Mudd et al. 2008; Chen et al. 2015). RNase J is unique since it has endo- and exonuclease activity and is strongly upregulated by light, although its mode of action remains to be studied in detail (Chen et al. 2015; Halpert et al. 2019).

After the cleavage of polycistronic into monocistronic precursor mRNAs, the RNA is stabilized, and introns are spliced out. Introns in plastids have lost the ability of self-splicing and depend on nuclear and plastid-encoded splicing factors (summarized in Zeng et al. (2022).

Several posttranscriptional RNA processing steps of nuclear genes, such as splicing and editing, are controlled by light. The expression of RNA splicing factors is upregulated during de-etiolation via photoreceptors, affecting splicing efficiency. Different *PhANG* transcripts have an altered and higher splicing rate in the light compared to darkness (Hu et al. 2024). In addition, key players in nuclear light signaling pathways, such as CONSTITUTIVE PHOTOMORPHOGENESIS1 and PIFs (Box I), repress the expression of RNA editing factors. During de-etiolation, this repression is relieved, leading to changes in RNA editing rates that support chloroplast development (Hu et al. 2024).

Although light clearly affects plastid transcription and RNA editing, due to the relatively stable transcripts, it is argued that protein homeostasis in the plastid is predominantly regulated at the translational level (Gao et al. 2023). Plastid-encoded genes are translated by plastid ribosomes that are similar to bacterial ribosomes. However, their function is more complex and requires additional translational factors, both plastid- and nuclear-encoded (Manuell et al. 2007; Sun and Zerges 2015). The initiation of translation is the most tightly controlled step (Gao et al. 2023). Ribosome binding to specific transcripts is light-dependent and selective, independent of transcript abundance (Liu et al. 2013). During de-etiolation, light induces the translation of proteins involved in photosynthesis and translation via increased ribosome occupancy (Liu et al. 2012). In maize grown under day/night cycles, a transition from dark to light does not increase ribosome binding to the RNA but increases the translation elongation speed (Chotewutmontri and Barkan 2018). Additionally, in barley, light increases the translation of *psaA*, *psaB*, *psbA*, and *RbcL* from transcripts already present in the dark without an increased occupancy of initiation sites (Klein et al. 1988; Kim et al. 1994; Edhofer et al. 1998). During translation, specific pausing sites in some photosystems I and II (PSI and PSII, respectively) subunit RNAs allow the binding of chlorophyll and other cofactors (Kim et al. 1994; Gawroński et al. 2018). Correct folding and protection from degradation of these apoproteins requires the binding of chlorophyll, which accumulates in the light (discussed below in more detail) (Eichacker et al. 1996; Nickelsen and Rengstl 2013). The co-translational translocation adds an extra layer of control for plastid-encoded membrane-localized proteins. To ensure proper folding and insertion of the proteins into the thylakoid membrane, in maize, approximately half of the membrane-localized proteins are inserted into the membrane during translation (Zoschke and Barkan 2015).

Protein functionality is strongly regulated by PTMs. In plastids, PTMs affect both chloroplast-encoded and nuclear-encoded proteins. In adult plants, a dark-to-light switch modifies up to 1,700 sites in chloroplast proteins. Most of these are either required for photosynthesis or induced by the onset of photosynthesis (Giese et al. 2023). PTMs can fine-tune specific enzymatic steps in the tetrapyrrole pathway to quickly respond to the environment. For example, phosphorylation of GENOMES UNCOUPLED4 in darkness prevents protochlorophyllide overaccumulation (Richter et al. 2016). When photosynthesis is established, reversible phosphorylation of LHCII maintains the energy balance between PSI and PSII (Minagawa 2011).

Protein homeostasis in chloroplasts is also regulated by protein degradation. Approximately 20 proteases are present in the chloroplast. For example, stroma-located Clp is a major degradative complex, essential for proteohomeostasis. It is involved in quality control and in the degradation of proteins involved in the tetrapyrrole pathway (Nishimura et al. 2016). The chloroplast-encoded subunit ClpP1 is edited less efficiently when etiolated seedlings are transferred to light, which reduces Clp protease activity. Through this mechanism, light decreases protein degradation, resulting in the accumulation of proteins required for chloroplast development (Hu et al. 2024).

## Light brings color to the plastids: pigment accumulation

**Figure 2:**
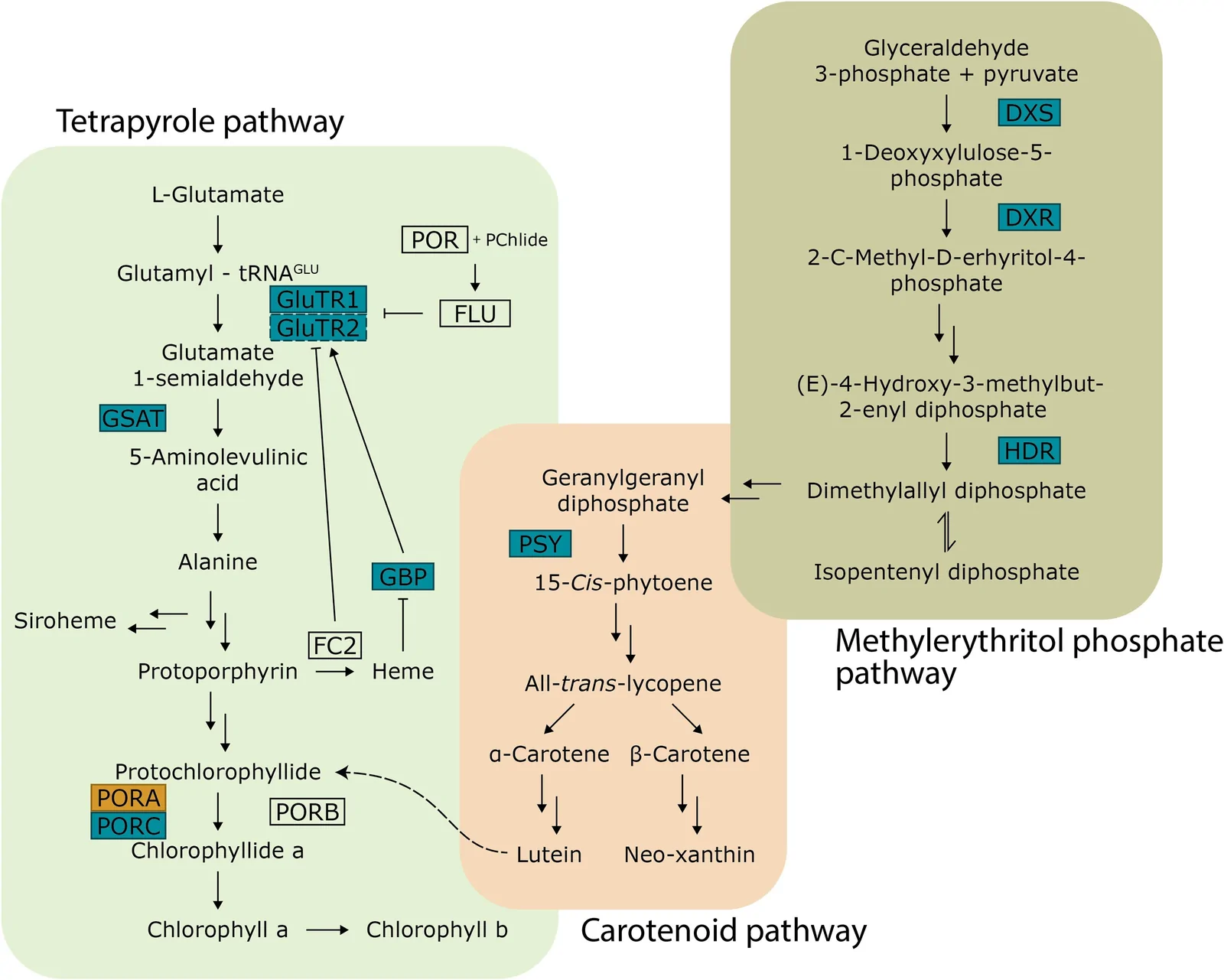
Simplified schematic representation of tetrapyrrole, methylerythritol phosphate (MEP), and carotenoid biosynthesis pathways and the light-regulated mechanisms during de-etiolation. Light promotes the accumulation of different pigments, such as chlorophylls and carotenoids. The conversion from protochlorophyllide to chlorophyllide is strictly light-dependent because of the light activation of POR. Additionally, the flux through these pathways is carefully regulated to ensure proper pigment production during greening. This regulation is done on the gene level (*HEMA1* [encoding GluTR1], *glutamate-1-semialdehyde-2,1-aminotransferase* [*GSAT*], *PORA*, and *PORC*, and *phytoene synthase* [*PSY*]), the protein level (GluTR1, GluTR2, and GBP), or both (1-deoxy-D-xylulose 5-phosphate synthase [DXS], 1-deoxy-D-xylulose 5-phosphate reductoisomerase [DXR], and 4-hydroxy-3-methylbut-2-enyl diphosphate reductase [HDR]). A blue box indicates upregulation upon light exposure, whereas orange indicates downregulation. The dashed line represents a regulatory mechanism that is not yet fully understood. See online supplementary material for a color version of this figure.

During the dark-to-light transition, protein expression is tightly synchronized with pigment synthesis. Chloroplasts contain 2 important classes of pigments: carotenoids and tetrapyrroles (Fig. 2). Carotenoids fulfill a photoprotective role during light transition by functioning as antioxidants (Gray et al. 2003; Rodríguez-Villalón et al. 2009b). Tetrapyrroles, such as chlorophylls, heme, and siroheme, are crucial metabolites in various processes (Tanaka et al. 2011). Chlorophylls are essential photosynthetic pigments, involved in light-harvesting, energy transfer, and charge separation (Kato et al. 2020). Siroheme is crucial for sulfur and nitrogen assimilation, and heme is essential for, among others, redox reactions in the electron transport chain (Tripathy et al. 2010; Brzezowski et al. 2015).

Tetrapyrrole biosynthesis occurs completely in the plastid (Shimizu and Masuda 2021) and starts with a common precursor pathway that splits into 3 branches, responsible for the production of siroheme, heme, and chlorophyll (van Veen et al. 2025) (Fig. 2). It starts with the L-glutamate to 5-aminolevulinic acid (ALA) conversion, catalyzed by Glutamyl-tRNA reductase (GluTR) (Tanaka et al. 2011) (Fig. 2). Arabidopsis has 3 GluTR encoding genes, of which *HEMA1* (GLUTR1) is the only 1 expressed in photosynthetic tissues and controlled by light (Ilag et al. 1994; Schmied et al. 2018). *HEMA2* (encoding GluTR2) is expressed at low levels in darkness, to allow for continued ALA synthesis, while suppression of GluTR1 prevents protochlorophyllide overaccumulation (Czarnecki et al. 2011; Apitz et al. 2014). *HEMA3* is considered a pseudogene (Matsumoto et al. 2004). Glutamate-1-semialdehyde-2,1-aminotransferase (GSAT) catalyzes the second step of the pathway and is upregulated during photomorphogenesis (Fig. 2) (Ilag et al. 1994; McCormac et al. 2001; Schmied et al. 2018).

In the absence of light, the chlorophyll branch halts at protochlorophyllide. The conversion from protochlorophyllide to green chlorophyll pigments is the only step in the tetrapyrrole pathway that fully depends on light. Consequently, protochlorophyllide accumulates in dark-grown seedlings. During the transition to photoautotrophy, protochlorophyllide absorbs a photon (<700 nm) that is used for its reduction (Sperling et al. 1998; Dietzek et al. 2004). This reaction is catalyzed by PROTOCHLOROPHYLLIDE OXIDOREDUCTASE (POR) and uses NADPH as a hydride donor to produce chlorophyllide (Archipowa et al. 2018). Angiosperms depend fully on the light-activated PORs for chlorophyll production (Gabruk and Mysliwa-Kurdziel 2020). Arabidopsis encodes 3 PORs: PORA, PORB, and PORC (Masuda and Takamiya 2004). *PORA* is highly expressed in etiolated seedlings but decreases rapidly upon light exposure. On the contrary, *PORC* is not expressed in darkness but increases in light. The expression of *PORB* is not affected by light (Su et al. 2001; Masuda and Takamiya 2004) (Fig. 2).

Precursor levels in the chlorophyll branch are carefully regulated because high levels of free intermediates can cause oxidative stress during light transition, mainly by forming singlet oxygen (^1^O_2_) (op den Camp et al. 2003; Tripathy and Oelmüller 2012). Protochlorophyllide and other intermediates in the chlorophyll synthesis pathway bind to proteins such as POR or LHCs in the photosystems to prevent excessive ROS production and subsequent oxidative stress upon light exposure (Triantaphylidès and Havaux 2009; Wang and Grimm 2021).

Additionally, the accumulation of downstream intermediates suppresses ALA synthesis in the dark to prevent oxidative stress upon light exposure (Apitz et al. 2014). This feedback regulation occurs in both the chlorophyll and the heme branches. For example, posttranslational regulation of GluTR1 is directed by FLUORESCENT IN BLUE LIGHT (FLU) (Meskauskiene et al. 2001; Hou et al. 2019). In the dark, the buildup of POR bound to protochlorophyllide promotes complex formation of FLU/POR/Mg-protoporphyrin IX monomethylester oxidative cyclase (CHL27) and binding to GluTR, thereby increasing GluTR turnover and suppressing ALA synthesis (Kauss et al. 2012; Schmied et al. 2018). Loss of FLU results in an overaccumulation of protochlorophyllide, and upon illumination, light energy is transferred from protochlorophyllide to oxygen, yielding singlet oxygen (op den Camp et al. 2003). *Flu* seedlings bleach and die upon light exposure (Meskauskiene et al. 2001). This necrotic response might be caused by the singlet oxygen itself, but the strong nuclear reprogramming upon singlet oxygen in this mutant can also be signaled via EXECUTER1 and EXECUTER2, since e*x1/ex2/flu* triple mutants have lost almost all nuclear responses as well as the necrotic phenotype upon singlet oxygen stress (Lee et al. 2007).

Another feedback loop that regulates the tetrapyrrole pathway acts via the binding of heme to, and thereby inhibiting, GluTR-binding protein (GBP) (Richter et al. 2019). This releases the protective function of GBP on GluTR, and GluTR is degraded (Apitz et al. 2016). FLU regulates GluTR independently of GBP (Goslings et al. 2004). Another regulator of ALA production is the heme-producing enzyme FERROCHELATASE 2. Like FLU, FERROCHELATASE 2 can interact with the POR-GluTR complex and suppress ALA synthesis (Fan et al. 2023). Overall, the tetrapyrrole pathway is tightly regulated at different levels to ensure a rapid acclimation to the light, while preventing phototoxicity (Cheminant et al. 2011).

Carotenoids form another class of pigments important in chloroplast development. They function as antioxidants for photoprotection during etioplast-to-chloroplast development and are important in photosystem assembly and light-harvesting (Domonkos et al. 2013). The methylerythritol phosphate pathway is the main pathway supplying carotenoid precursors in etiolated seedlings (Rodriguez-Villalon et al. 2009a). This plastid-localized pathway is upregulated under the influence of light (Rodríguez-Concepción 2006). Transcription and protein synthesis of rate-limiting enzymes, such as 1-deoxy-D-xylulose 5-phosphate synthase, 1-deoxy-D-xylulose 5-phosphate reductoisomerase, and 4-hydroxy-3-methylbut-2-enyl diphosphate reductase, increase during de-etiolation via direct regulation by PIFs and ELONGATED HYPOCOTYL5 (HY5) in the nucleus (Box I) (Chenge-Espinosa et al. 2018).

The first committed step in carotenoid synthesis is the formation of 15-cis-phytoene from 2 geranylgeranyl diphosphate molecules. Besides its impact on the upstream methylerythritol phosphate pathway, light tightly regulates carotenoid synthesis itself (Fig. 2). The formation of 15-cis-phytoene is catalyzed by the enzyme PHYTOENE SYNTHASE (PSY), of which expression is strongly suppressed by PIFs in darkness and induced by HY5 in light (Box I) (Rodriguez-Villalon et al. 2009a; Toledo-Ortiz et al. 2014). Moreover, PSY activity is regulated through alternative splicing and protein-protein interactions (Zhou et al. 2022; Hou et al. 2024). PSY promotes the accumulation of cis-carotenes, which can produce an unidentified apocarotenoid signal (Cazzonelli et al. 2020). This signal fine-tunes PSY activity to suppress plastid development, through upregulation of PIF3 and downregulation of HY5. In darkness, cis-apocarotenoid signal accumulation provides an alternative mechanism for regulating *PhANG* expression and carotenoid levels in etioplasts (Hou et al. 2024).

## Light directs structural changes in the plastid

Plastid lipids are important in the etioplast-to-chloroplast transition. Studies in barley have shown that the lipid composition changes already during the first minutes after illumination (Selldén and Selstam 1976). The PLBs in etioplasts, a buildup of protochlorophyllide in complex with POR, NADPH, and carotenoids, are embedded in the scaffolding lipid matrix (Bykowski et al. 2020). These galactolipids are essential for rapid light acclimation when the thylakoid membrane is formed. Next to the PLB, the galactolipids are stored in the plastoglobuli in etioplasts, which decrease in number upon thylakoid development (Fig. 1) (Rottet et al. 2015).

The correct development of the thylakoid membrane is crucial for the primary task of chloroplasts: photosynthesis. Here, the lipids serve not only as a scaffolding matrix for important proteins; they also regulate *PhANG* expression, are involved in chlorophyll synthesis, and allow for chloroplast compartmentalization (essential for functional photosynthesis in the light) (Fujii et al. 2019; Hölzl and Dörmann 2019). The lipids needed for this membrane, and the majority of the plant lipids, are produced in the chloroplast itself (Hölzl and Dörmann 2019).

The 4 most abundant plastid lipids that make up the PLB, and later the inner plastid and thylakoid membranes, are monogalactosyldiacylglycerol (MGDG), digalactosyldiacylglycerol (DGDG), sulfoquinovosyldiacylglycerol, and phosphatidylglycerol (Selstam and Sandelius 1984; Hölzl and Dörmann 2019). Both MGDG and DGDG oligomerize with PORA and PORB and form the typical hexagonal structures of the PLB (Gabruk et al. 2017). These polyoligomers facilitate excitation energy transfer and thus efficient catalysis of protochlorophyllide to protochlorophyll at the onset of light (Gabruk et al. 2017). The interaction of PORA and PORB with phosphatidylglycerol, sulfoquinovosyldiacylglycerol, and MGDG increases the NADPH binding affinity to POR, leading to higher POR activity (Gabruk et al. 2025). Upon light transition, POR catalyzes the protochlorophyllide to chlorophyllide conversion and thereby disperses the PLB (Gabruk and Mysliwa-Kurdziel 2020). Additionally, the incoming light alters the internal membrane structure by altering the lipid composition.

The different lipid ratios strongly determine the plastid membrane's secondary structure and are therefore tightly regulated. The inverted hexagonal structures formed when MGDG levels are high are partly responsible for the characteristic honeycomb-like structure of PLBs in etioplasts (Shipley et al. 1973; Selstam and Sandelius 1984; Demé et al. 2014). The transition to light causes a decrease in the MGDG/DGDG ratio, which coincides with the reorganization from the hexagonal to the lamellar structures of the thylakoid membranes (Demé et al. 2014; Rocha et al. 2018; Pipitone et al. 2021). This reorganization occurs within nanoseconds without external energy. Additionally, the light-induced protochlorophyllide to chlorophyllide conversion promotes the dissociation of POR-chlorophyllide complexes from the PLB and initiates the formation of the stroma lamellae and grana stack structures (Solymosi and Schoefs 2010). The full disappearance of PLBs in the light happens within 4 h, and afterwards the MGDG/DGDG ratio increases again. In the first 24 h of seedling development, the MGDG and DGDG are mainly synthesized via the endoplasmic reticulum pathway, but afterwards, the plastid-localized pathway becomes more important (Pipitone et al. 2021). The exact regulation of the lipid ratio shifts by light remains unclear. However, during de-etiolation, HY5 accumulates and promotes the expression of genes encoding MGDG synthase (*MGD1*) and DGDG synthase (*DGD1*) (Kobayashi et al. 2014). To underline the importance of the lipids, knock-out of *MGD1*, the gene encoding an enzyme that catalyzes the production of MGDG, causes detrimental effects in both etioplast and chloroplast structure and results in bleached seedlings (Kobayashi et al. 2007; Fujii et al. 2019). Additionally, *dgd1-*deficient Arabidopsis seedlings, which fail to synthesize DGDG from MGDG, form no prothylakoids in etioplasts and are strongly defective in thylakoid membrane formation after the light transition (Fujii et al. 2019).

Incorporating proteins into the thylakoid membrane allows for planar stabilization, despite the increased MGDG (Selstam and Sandelius 1984; Kirchhoff et al. 2002). Approximately 70% of the chloroplast membrane area is covered with proteins (Kirchhoff et al. 2002). Some proteins, such as CURVATURE THYLAKOID 1A (CURT1A), are specifically involved in membrane remodeling (Armbruster et al. 2013). CURT1A localizes on the outside of the PLB, where, upon light exposure, the decay happens, and the grana discs are formed (Liang et al. 2022). In light, CURT1A localizes to the highly curved margins of the grana discs and facilitates this strong bending (Armbruster et al. 2013; Liang et al. 2022). Later, the presence of LHCII helps to stabilize the grana stacks (Albanese et al. 2020). As a consequence, plants with LHCII complex defects show strongly impaired grana stacking (Cui et al. 2010). Even in an artificial system with only lipids and LHCII, high LHCII causes stacking in the proteoliposomes, resembling grana stacks (Wilson et al. 2022). However, how the different forces between the grana stacks determine the final structure of these discs is a point of discussion (Ostermeier et al. 2024).

Lipidic carotenoids also contribute to thylakoid formation. Light exposure triggers the accumulation and ratio of the different carotenoids, which determines the fluidity of the thylakoid membrane. This fluidity is essential for proper grana stacking and the spatial organization of membrane-localized photosynthetic proteins. When the ratio between lutein and carotenoid becomes too high, the membrane stiffens, which results in reduced grana stacking (Bykowski et al. 2021).

Depending on the light conditions and species, forming the final internal structure of chloroplasts takes up to a couple of days (Adam et al. 2011). For Arabidopsis, the complete process happens within 24 h after transitioning to light (Pipitone et al. 2021). During thylakoid membrane formation and the accumulation of carotenoids, chlorophylls, and photosynthetic proteins, chloroplasts become active photosynthesizing organelles. All these processes must be orchestrated to form the lumen; the closed compartment of the thylakoid membrane where a proton potential can be maintained, and which hosts all protein supercomplexes involved in photosynthesis, while maintaining the correct membrane fluidity to allow adaptation to variable light conditions (Kirchhoff 2019; Matzner et al. 2023).

## Light, chloroplast, action

The structural establishment phase, in which etioplasts adapt to the light by remodeling the PLB to the thylakoid formation and enabling photosynthesis, is followed by the greening and the proliferation phases (Pipitone et al. 2021; Cackett et al. 2022), during which chloroplasts become fully photosynthetically active. In Arabidopsis seedlings, it takes approximately 14 h of continuous white light to establish a fully functional photosynthetic machinery (Pipitone et al. 2021).

Directly after light exposure, the developing etioplasts start using photosynthetically active radiation (λ = 400–700 nm). In isolated etioplasts from beans, PSI activity can be detected as quickly as 10 min after light exposure and before electron transport starts, because of the ability of cyclic electron flow in PSI (Gyldenholm and Whatley 1968; Bertrand et al. 1988). Light is absorbed by LHCs, made up of proteins, carotenoids, and chlorophyll *a* and *b* (Pan et al. 2013). PSI and PSII are located preferentially in the stroma lamella and the grana stack, respectively (Wietrzynski et al. 2020). The lipid matrix allows for proper spacing of the photosynthetic complexes, but the lipids are an integral part of PSI, PSII, and cytochrome *b6f* (Kobayashi et al. 2016). After PSI and PSII assembly, chloroplasts start to photosynthesize; light drives the formation of molecular oxygen, free electrons, and protons at PSII and the electron transport chain from PSII to PSI. Splitting of water and the electron transport chain create a proton gradient over the thylakoid membrane, which fuels ATP production by ATPases (Mitchell 1966). The electrons are used to produce NADPH. ATP and NADPH provide energy for the carbon reaction that converts CO_2_ and H_2_O into glucose and O_2_, the energy sources to drive further plant development (extensively reviewed by Stirbet et al. (2020).

Chloroplast biogenesis and assembly of the photosynthetic machinery during the etioplast-to-chloroplast transition are coordinated by multiple photoreceptors, including phytochromes, cryptochromes, phototropins, and UVR8 (Seluzicki et al. 2017) (Box I). Nevertheless, light-induced promotion of photosynthesis-related gene expression can be compromised by specific light conditions after chloroplasts are formed. For example, under shaded, far-red rich conditions, phytochromes are partially reverted to their inactive form, leading to increased PIF activity that represses photosynthetic gene expression (Leivar and Quail, 2011). Similarly, shade promotes the CONSTITUTIVE PHOTOMORPHOGENESIS1–mediated degradation of HY5, resulting in repression of genes for pigment biosynthesis, LHCs, photosystem core proteins, and RUBISCO (Toledo-Ortiz et al. 2014), as well as genes involved in fine-tuning PSI/PSII stoichiometry and light capture (Toman et al. 2024). In addition, metabolic feedback integrates chloroplast and photoreceptor signaling (Box III). Sugars and organic acids regulate *PhANG* expression through the HY5/PIF module, coordinating pigment biosynthesis with photosystem assembly and stress responses (Toledo-Ortiz et al. 2014; Mankotia et al. 2024). Elevated sugar levels suppress phytochrome–HY5 signaling to restrict growth, while carbon and nitrogen depletion activate HY5 (Mankotia et al. 2024).

Besides photosynthesis, chloroplasts fulfill many other important tasks. They are storage organs for starch granules, which serve as energy reserves during the night (Smith and Stitt 2007). Chloroplasts are involved in nitrate and sulfur assimilation, which are used for amino acid biosynthesis, and as alternative ways to release excess energy during photosynthesis (Hoff et al. 1994; De Bont et al. 2022; Gilad et al. 2025). Chloroplasts house the synthesis of nucleotides, isoprenoids, and hormones (Chen et al. 2018). Also, chloroplasts are involved in sensing of and adaptation to environmental stresses (van Veen et al. 2025). Notably, the chloroplast is not the end station of the plastid development. They can further develop into plastids with specific functions, such as carotenoid-accumulating chromoplasts or starch-packed amyloplasts. These transitions are largely reversible and influenced by the plant's environment (Sadali et al. 2019).

## Concluding remarks

Many processes rapidly adapt when a seedling emerges from the soil and is exposed to light. Upon illumination, plastid and nuclear processes act in concert to establish a functional chloroplast. Gene expression, posttranscriptional and translational changes, and pigment biosynthesis are essential to build the core complexes needed for photosynthesis. Most of these processes are proven to be light-regulated, via nuclear signaling or the direct impact of light on chlorophyll synthesis. Nevertheless, for many steps in plastid development, the exact involvement of light remains unclear. Moreover, although biogenic retrograde signals that mediate feedback communication between developing plastids and the nucleus have been studied for a long time, the signaling components remain largely unknown. Lipids provide the scaffolding structure for the nuclear- and plastid-encoded protein complexes and form the membranes that separate the stroma from the lumen. These structural features allow chloroplasts to fulfill their core task: photosynthesis, which marks the end of heterotrophic growth.

Be it through photoreceptor signaling, retrograde pathways, or circadian regulation, light plays an essential role in regulating a broad range of processes during the development of etioplasts into chloroplasts. The elegant and complicated entanglement of the different nuclear and plastid processes allows for a smooth adaptation to an ever-changing environment and gives plants the robustness to survive under suboptimal conditions.

Advances box

- Links between light signaling cascades and photosynthesis have led to synergistic approaches in both fields of study.
- Light impacts chloroplast establishment via local and inter-organellar pathways
- Plastid structure and functions are linked, as exemplified by the interactions between plastid lipids and POR enzymes, which form the internal structures of etioplasts and promote POR activity by increasing NADPH binding affinity.
- Phytochrome-controlled anterograde signals reprogram plastid transcription during chloroplast establishment.
- New techniques focusing on structural dynamics allow fine mapping of plastid development over time and reveal the 2 phases of chloroplast development.

Outstanding questions box

- How does light of different wavelengths contribute to the etioplast-to-chloroplast transition?
- How is light involved in the establishment of other plastid types?
- What is the impact of abiotic stresses on the etioplast-to-chloroplast development?
- How do different plastid-to-nucleus retrograde signals drive plastid development?
- What are the effects of plastid-derived retrograde signals on plant development during the transition to light?
- How does the circadian clock mechanistically regulate plastid transitions and function?
- Do organ- or cell-type-specific nuances exist for the etioplast-to-chloroplast transition?

## Data Availability

No new data is generated for this review.
